# Oral Manifestations of Crohn's Disease: A Case Report and Review of the Literature

**DOI:** 10.1155/2015/830472

**Published:** 2015-07-09

**Authors:** Victoria L. Woo

**Affiliations:** Oral and Maxillofacial Pathology, School of Dental Medicine, University of Nevada, Las Vegas, NV 89117, USA

## Abstract

Crohn's disease (CD) is an inflammatory disorder of the gastrointestinal tract that is likely caused by an inappropriate mucosal inflammatory response to intestinal bacteria in a genetically predisposed host. The lesions of CD can involve any region of the GI tract as well as extraintestinal sites such as the skin, joints, and eyes. The most common presenting symptoms are abdominal pain and prolonged diarrhea associated with fevers, fatigue, and malaise. Delayed growth and failure to thrive may also be observed in pediatric patients. Oral manifestations of CD are known as oral CD and may precede GI involvement, thus serving as early markers of this condition. We describe a 6-year-old male who presented with oral lesions as his initial manifestation of disease and review the current literature pertaining to oral CD.

## 1. Introduction

Crohn's disease (CD) is an immune-mediated disorder of the gastrointestinal (GI) tract which, along with ulcerative colitis, comprises the two major subsets of the inflammatory bowel disease (IBD). The underlying etiology is poorly understood but likely involves defects in mucosal immunity and intestinal epithelial barrier function in a genetically susceptible individual, leading to an inappropriate inflammatory response to intestinal microbes [1–3]. The lesions of CD can involve any portion of the alimentary tract from the mouth to anus [4, 5]. Extraintestinal sites such as the skin, joints, and eyes may be affected as well. The most common presenting symptoms are periumbilical abdominal pain and diarrhea associated with recalcitrant fevers, malaise, fatigue, and anorexia [1, 4, 5]. Oral involvement is identified in up to 80% of patients [4, 6, 7] and may precede GI involvement in some cases. We describe a pediatric patient whose initial presentation of CD was multifocal gingival erythema and swelling.

## 2. Case Report

A 6-year-old male presented with his mother for evaluation of painful and bleeding gingiva. The patient's mother reported that the gingival changes began seven months ago and that the onset was not associated with any identifiable inciting events, including mechanical, thermal, and chemical trauma; dietary changes; use of new dental hygiene products; or exposure to cinnamon-containing products or foodstuffs. She also denied a history of fever, malaise, and GI symptoms. The patient had seen his pediatrician two weeks prior and undergone a complete blood count (CBC) and metabolic panel, which revealed no abnormalities. A thorough dental prophylaxis had also been performed one week prior with no significant improvement in the appearance of his gingiva.

The patient's medical history was significant for asthma, for which he was using mometasone furoate inhaler once daily and albuterol sulfate inhaler on an as-needed basis. His mother stated that he took special care rinsing his mouth after use of the steroid inhaler as directed by his pediatrician. His review of systems and family history were otherwise unremarkable for cardiovascular, endocrinologic, GI, genitourinary, musculoskeletal, hematologic, and neurologic disorders.

**Figure 1:**
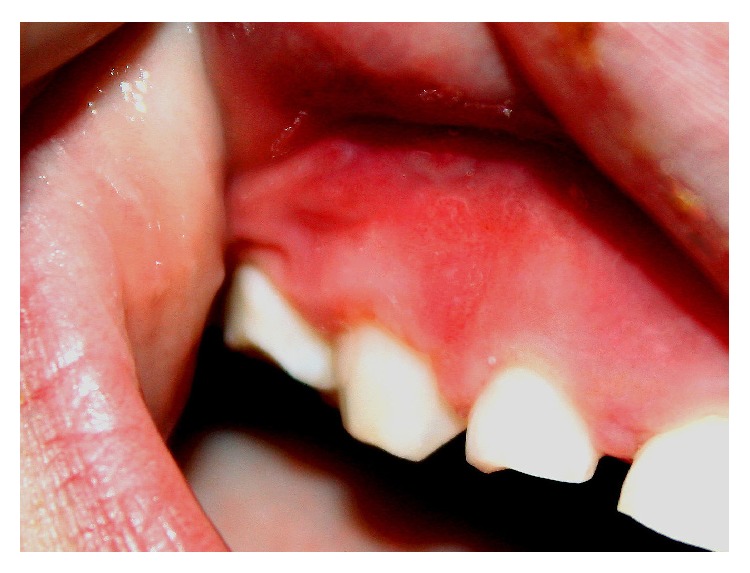
Mild mucosal erythema of the right anterior maxillary gingiva.

**Figure 2:**
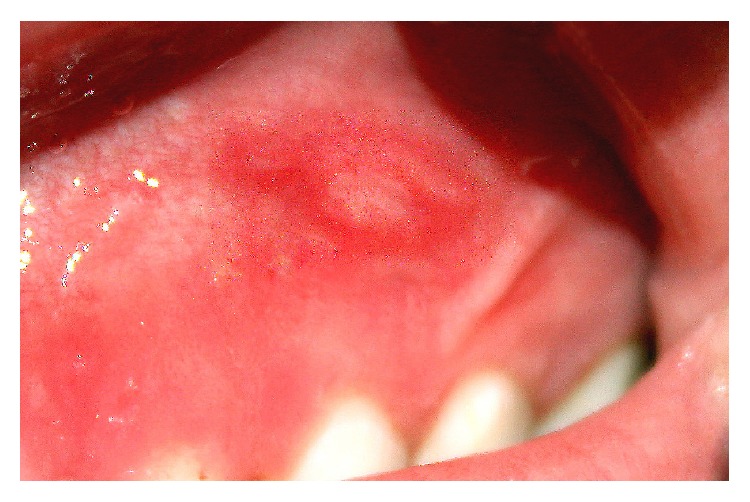
A demarcated area of mucosal erythema involving the left anterior maxillary alveolar mucosa.

**Figure 3:**
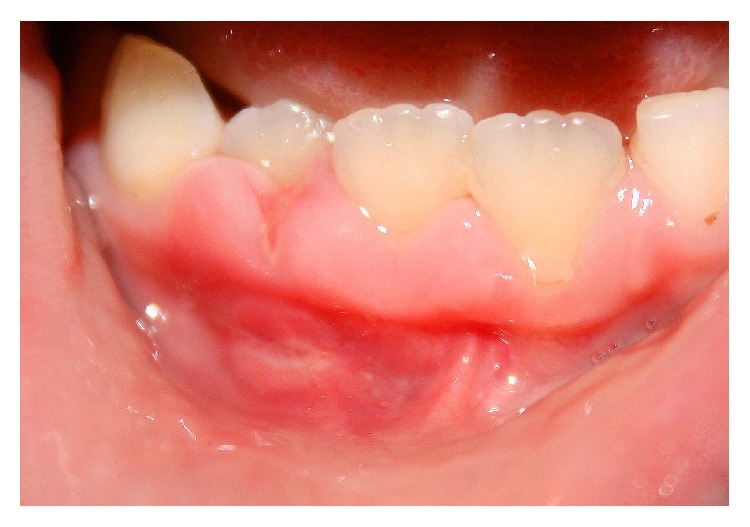
Nodular swellings of the interdental papillae between the right permanent mandibular central and lateral incisors and primary canine. An ulceration of the free gingival margin between the incisors is seen. Also noted is a linear ulceration with hyperplastic margins involving the alveolar mucosa.

Intraoral examination revealed a very subtle area of mucosal erythema involving the gingiva buccal to the right primary maxillary molar, canine, and lateral incisor (Figure 1) and a more well-defined area of erythema involving the alveolar mucosa facial to the left primary maxillary central and lateral incisors and canine (Figure 2). Also noted were multiple mucosal-colored swellings and ulcerations of the gingiva facial to the right permanent mandibular central and lateral incisors and primary canine (Figure 3). Mild tenderness was elicited on gentle palpation of the affected sites. The remainder of the oral mucosal examination was within normal limits. No bone loss was evident on radiographic examination.

**Figure 4:**
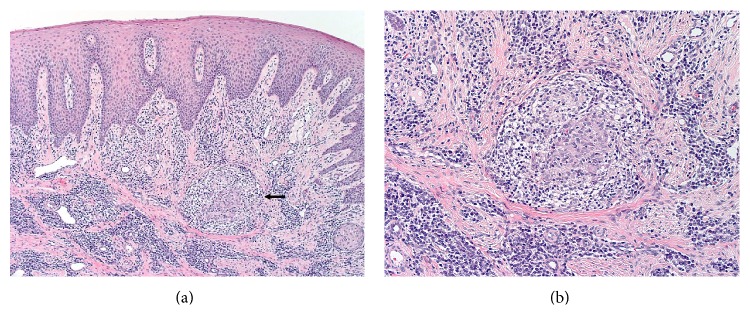
Histopathologic images of the right mandibular gingiva. (a) Low-power view showing stratified squamous epithelium with scattered intraepithelial lymphocytes (exocytosis). The underlying fibrous connective tissues are characterized by a patchy chronic inflammatory cell infiltrate and an isolated granuloma (arrow) (hematoxylin and eosin, 40x). (b) High-power view showing a well-defined, noncaseating granuloma composed predominantly of epithelioid histiocytes and lymphocytes (hematoxylin and eosin, 100x).

As requested by the referring periodontist, biopsy of the affected mandibular gingiva was performed. Microscopic examination revealed curved portions of oral mucosa surfaced by spongiotic stratified squamous epithelium with evidence of mild inflammatory cell exocytosis; beneath the epithelium, there was a proliferation of fibrous connective tissue with a patchy infiltrate of lymphocytes and plasma cells (Figure 4(a)). In isolated areas, there were well-formed, noncaseating granulomas composed of epithelioid histiocytes and lymphocytes (Figure 4(b)). Periodic Acid-Schiff (PAS), Grocott-Gomori's methenamine silver (GMS), and Acid-Fast Bacilli (AFB) stains were performed to rule out deep fungal infections and mycobacterial infections such as tuberculosis and leprosy as causes of the granulomas. All stains were negative. Examination of the biopsy under both light and polarized light microscopy did not reveal foreign body material. Hence, a diagnosis of granulomatous inflammation was rendered with a comment pertaining to possible etiologies such as Crohn's disease, orofacial granulomatosis, and sarcoidosis.

The patient was provided with a prescription for a mild topical steroid rinse and instructed to rinse and spit four times daily for 14 days. He was also referred to his pediatrician for additional evaluation to rule out for Crohn's disease and other systemic conditions associated with granulomatous inflammation, including sarcoidosis. The patient returned for follow-up three weeks later and demonstrated a modest reduction in the erythema of the maxillary gingiva and swelling of the mandibular gingiva. The patient's mother also reported less pain and bleeding during brushing. The patient was again advised to pursue diagnostic evaluation for systemic causes of granulomatous inflammation. Following evaluation by his pediatrician, he was referred to a pediatric gastroenterologist for consultation. The patient subsequently underwent endoscopic examination and biopsy which showed acute inflammation of the intestinal mucosa. A diagnosis of early Crohn's disease was made. The patient was placed on mesalamine, an anti-inflammatory aminosalicylate, and azathioprine, an immunosuppressant. At one-year follow-up by phone, the patient's mother reports that he continues to respond well to his medications. She states that his oral lesions have completely resolved and that he is currently free of intraoral and GI symptoms.

## 3. Discussion

CD is a multisystem, inflammatory disorder with a complex etiologic basis that is believed to involve an interplay of genetic, immunologic, and environmental factors [1]. It has been postulated that changes in the immune system and exposure to environmental risk factors are necessary triggers of disease [1]. The increasingly accepted theory is that CD is the result of an inappropriate mucosal inflammatory response to intestinal bacteria in a genetically predisposed host [2, 3]. Although a specific organism has yet to be consistently identified in CD patients [8, 9], the presence of bacteria appears to be an obligatory event in the pathogenesis of this disorder. This is supported by* in vivo* murine models of CD, in which the induction of mucosal inflammation is dependent on microbial stimulation [4]. As a corollary, it has also been shown that inflammation does not occur in mice raised in a bacteria-free environment [1, 4]. Although similar observations have been reported in human studies, the causative role of bacteria in these investigations remains speculative. It is noteworthy that the microbiota in the intestine is complex and consists of organisms that can potentially exert pro- and anti-inflammatory effects [4]. The shift towards an inflammatory state in CD is believed to be caused by alterations in the intestinal flora and the host's mucosal response, which is influenced by both genetics and immunity [4].

The role of genetics in the pathogenesis of CD was suggested early on with recognition of familial clustering and twin concordance among affected patients [4, 9–12]. A positive family history remains the most important independent risk factor for developing CD to date [4, 13]. More recently, genome-wide analysis studies have revealed more than 30 loci associated with CD [1, 14, 15]. Of significance may be the genetic polymorphisms that alter adaptive immunity and the mutations associated with inadequate surveillance of bacteria by the intestinal mucosa [16–20]. The cumulative effect of these genetic aberrations may be the development of abnormal immune tolerance to intestinal antigens [21, 22]. The inappropriate mucosal inflammatory response that ensues is likely a result of immune system dysregulation. Immunologic mechanisms proposed to be involved in CD include impairment of the innate immune system, leading to a sustained proinflammatory environment in the intestines [1]; excessive activation and differentiation of T-cell subsets against mucosal antigens [23–25]; and aberrant cytokine secretion [26, 27]. In particular, the cytokine IFN-*γ* appears to play a key role in maintaining the inflammatory milieu in the intestine [4]. Such findings may be significant in the development of more targeted CD therapies [4].

Lastly, certain environmental factors have been implicated in the pathogenesis of CD. These include sociodemographic factors such as economic growth, rises in income levels, and residence in urban areas [28, 29]; geographic factors such as exposure to northern climates [9]; and lifestyle factors such as tobacco smoking, use of oral contraceptives, diet, and psychological stress [9, 28].

CD has a reported incidence 3.1 to 14.6 cases per 100,000 person-years in North America and exhibits a bimodal age distribution, the first peak occurring in early adulthood and the second peak at 50 to 70 years of age [4]. The most common sites of involvement at initial diagnosis are the terminal ileum, ileocecal valve, and cecum [9]. However, any region of the GI tract may be affected, including other areas of the small and large intestines as well as the upper GI tract and oral cavity [4]. The clinical symptoms of CD can vary from patient to patient and depend primarily on the location and behavior of the lesions, disease severity and activity, and the involvement of extraintestinal sites. The most common symptoms include periumbilical or lower right quadrant pain, nonbloody diarrhea of greater than six months in duration, and weight loss accompanied by low-grade fever, malaise, and fatigue [4, 9]. This constellation of findings is believed to represent a harbinger for disease, especially in children [4]. In addition to the aforementioned symptoms, pediatric patients may experience fever of unknown origin, arthralgia, decreased growth, and failure to thrive [9, 30]. Laboratory findings are often nonspecific but may show evidence of GI malabsorption (e.g., low albumin, calcium, folate, iron, and red blood cell count), elevated erythrocyte sedimentation rate (ESR), elevated platelet counts, anemia, and increased acute phase reactants such as C-reactive protein [31–34]. Typical of most immune-mediated disorders, CD follows a chronic, indolent course characterized by periods of relapse and remission. However, the chronic nature of the inflammation ultimately predisposes patients to local complications such as strictures, fistulas, intra-abdominal abscesses, and bowel obstruction [9, 31]. These complications often compromise long-term intestinal function and may require surgical correction with time [4]. Recurrence of CD following surgery is common and is promoted by a number of factors such as a history of penetrating disease, young patient age, ileocolonic disease, and cigarette smoking [35, 36]. Lastly, CD patients are at risk for developing dysplasia or adenocarcinoma of the small intestine or colorectal mucosa [37]. Periodic colonoscopic surveillance is therefore a crucial aspect of management [9, 38, 39].

CD is characterized by an array of findings on endoscopic and microscopic examination. In contrast to ulcerative colitis, the pathologic lesions of CD occur in a segmented and discontinuous distribution [4, 9]. Identification of such “skip” lesions—sharply demarcated areas of disease surrounded by completely normal mucosa [9]—is considered a cornerstone in the diagnosis of CD. Once diagnosed, the patient must then undergo further evaluation such as imaging studies to assess the location and extent of lesions as well as the presence of complications [40–42].

It has been estimated that approximately 47% of patients with IBD exhibit extraintestinal manifestations (EIMs) which most frequently involve the skin, eyes, joints, liver, biliary tract, and lungs [4, 9]. Interestingly, the presence of one EIM has been shown to predispose to development of additional EIMs [4]. Involvement of the oral mucosa, termed oral CD, has a widely disparate prevalence rate of 0.5 to 80.0% [4, 6, 7, 43–47] that is likely attributable to differences in study inclusion criteria [4, 48]. It may precede, occur concurrently, or follow the onset of abdominal symptoms [31]. Synchronous or metachronous observation of oral lesions is most commonly described, although Plauth et al. [45] reported oral CD as the presenting symptom in 60% of their patients. Oral lesions considered pathognomonic for CD include persistent lip swelling, cobblestoning of the oral mucosa, mucogingivitis, deep linear or serpiginous ulcerations surrounded by epithelial hyperplasia, and tissue tags or polyps (Table 1) [4, 5, 31, 49, 50]. These may be associated with pain, impairment of oral function, and psychosocial stress [45]. It is important to bear in mind that affected patients may not always present with these classic lesions and detection of oral CD can be challenging, particularly if the lesions are subtle or the patient is in early stages of disease. Other oral findings that may be seen include aphthous ulcerations, angular cheilitis, stomatitis, glossitis, and perioral dermatitis [4]. However, these are considered nonspecific for CD as they may represent primary manifestations of disease or occur secondary to nutritional deficiencies induced by intestinal malabsorption, dietary restriction, or medications [4, 50]. Oral CD often demonstrates a young age of presentation and is most frequently seen in adolescents and young adults [45]. Interestingly, Hussey et al. [51] reported that only 29% of the children in their study continued to harbor CD-specific oral lesions over a mean follow-up of 55 months and emphasized the need for timely recognition and biopsy in the pediatric population. Harty et al. [47] reported the presence of granulomatous inflammation in 100% of the biopsies performed in their study and reiterated the value of the easily accessible oral mucosa as a potential site for harvesting diagnostic material, especially in children.

The clinical differential diagnosis for oral CD in a pediatric patient is broad and encompasses a variety of conditions that may present with oral mucosal swellings, masses, and/or ulcerations as follows:

*Multifocal Mucosal Swelling and Cobblestoning of Mucosa*

 
Idiopathic orofacial granulomatosis
 
Neurofibromatosis
 
Multiple endocrine neoplasia (MEN) 2B/III
 
Multiple hamartoma syndrome (Cowden disease)
 
Multifocal epithelial hyperplasia (Heck's disease)
 
Amyloidosis.

*Ulcerations (Linear and Aphthous-Like)*

 
Recurrent aphthous stomatitis
 
Aphthous ulcerations associated with an underlying systemic disease including
   
celiac disease,
   
ulcerative colitis,
   
immunocompromised conditions (e.g., HIV and AIDS),
   
hematologic disorders (e.g., neutropenia, cyclic neutropenia, and leukemia),
   
nutritional deficiencies,
   
MAGIC syndrome (i.e., mouth and genital ulcers with inflamed cartilage),
   
PFAPA syndrome (i.e., periodic fever, aphthous stomatitis, pharyngitis, and cervical adenitis),
   
Behçet's syndrome.
 
Viral infections including
   
herpes simplex infection,
   
varicella zoster infection,
   
enterovirus infection (e.g., hand-foot-mouth disease and herpangina).
 
Traumatic ulcerations
 
Chemical or thermal burns
 
Pemphigus vulgaris
 
Mucous membrane pemphigoid
 
Erythema multiforme.

*Swellings and/or Ulcerations That Exhibit Granulomatous Inflammation on Microscopic Examination*

 
Foreign body reaction and foreign body gingivitis
 
Allergy
 
Orofacial granulomatosis, including Melkersson-Rosenthal syndrome and cheilitis granulomatosa
 
Sarcoidosis
 
Tuberculosis
 
Leprosy
 
Deep fungal infections (e.g., histoplasmosis, blastomycosis, paracoccidioidomycosis, and coccidioidomycosis)
 
Tertiary syphilis.

Diagnostic considerations prior to histologic examination include idiopathic orofacial granulomatosis; syndromes presenting with multiple mucosal swellings, such as neurofibromatosis, multiple endocrine neoplasia (MEN) 2B/III, and multiple hamartoma syndrome (Cowden disease); aphthous ulcerations in the presence or absence of an underlying systemic disease; and other vesiculoerosive diseases of various etiologies. The differential diagnosis for histologically confirmed oral granulomatous inflammation in a younger patient can be broadly subcategorized into disorders of localized or systemic etiology. Localized causes of granulomatous inflammation include a foreign body reaction (e.g., granulomatous gingivitis); allergic reaction to cinnamon or benzoate; and idiopathic orofacial granulomatosis, a diagnosis of exclusion that requires elimination of other defined causes of granulomatous inflammation. Systemic conditions associated with granulomatous inflammation include Crohn's disease, sarcoidosis, mycobacterial infections, deep fungal infections, and tertiary syphilis. Ultimately, the diagnosis of CD hinges on careful correlation between the patient's clinical history and findings on physical, radiographic, endoscopic, laboratory, and microscopic examinations.

The management of CD is dependent on the disease location and activity as well as the presence of complications [4]. The predominant classes of medications used in the treatment of CD include anti-inflammatory agents such as aminosalicylates and steroids, immunosuppressants or immunomodulators such as thiopurines and methotrexate, and biologic agents such as infliximab. The standard “step-up” protocol advocates use of oral corticosteroid therapy for patients with mild-to-moderate CD localized to the ileocecal region [52] and a combination of oral corticosteroids and immunosuppressants for patients with moderate-to-severe small bowel disease and relapsing or steroid-refractory disease [52–54]. Biologic agents are recommended for patients who do not respond to or cannot tolerate standard therapy and in whom corticosteroids are contraindicated [4]. Surgery is typically postponed for as long as possible as it is not considered curative and may be associated with a number of functional complications and recurrence of disease [4]. Recently, there has been a shift in favor of administering biologic agents such as infliximab in patients with newly diagnosed CD, a so-called “top-down” approach [55]. It is believed that introduction of biologics early in disease may disrupt the natural evolution of CD from the inflammatory stage to the advanced stages, which is generally less responsive to pharmacologic therapy and more often associated with the development of complications [4, 56]. Management is more complex in the pediatric population because of the potential impact of CD medications on growth and development. The overall goals of CD treatment in children are to achieve the best possible control of disease with the least adverse effects, to promote continued growth through nutrition, and to enable the child to maintain normal day-to-day activities such as attending school [57]. Although no standardized protocol exists, most clinicians follow a step-up approach of administering an aminosalicylate, antibiotics, and nutritional therapy, followed by the addition of corticosteroid, immunomodulatory, and biologic therapy as deemed necessary [57]. As with adult CD, there is early evidence to support the top-down approach but further studies are necessary before widespread implementation [57]. Exclusive enteral nutrition (EN), also known as tube feeding, is another therapeutic option in children with CD [58, 59]. Pain associated with oral CD can be managed with topical, systemic, or injectable steroids with or without the use of immunosuppressive agents such as azathioprine [31, 45]; however, oral lesions typically respond well to systemic treatment of intestinal CD [50].

In summary, we have described a 6-year-old male with oral lesions as his initial manifestation of CD. This case underscores the importance of recognizing the variable, sometimes subtle, presentation of oral CD and performing necessary diagnostic procedures, such as biopsy for histopathologic confirmation. Astute identification of oral lesions is key as studies have shown that only a minority of patients will continue to exhibit oral findings at follow-up [51]. In addition, it has been reported that the ability of physicians to identify oral CD was poor when a dentist's exam was used as a comparator [47]. The dental practitioner is therefore in a unique position to detect oral CD, which may be the only manifestation of occult disease in patients who are otherwise asymptomatic. This may lead to early diagnosis, timely treatment, and ultimately a better outcome in affected patients.

## Figures and Tables

**Table 1:** Oral manifestations of Crohn's disease.

Lesion	Site(s)	Characteristics
Persistent mucosal swelling	Lips, buccal mucosa	Labial enlargement, firm to palpation, typically painless
Cobblestoning of mucosa	Buccal mucosa, vestibule	Mucosal edema with or without fissuring
Mucogingivitis	Attached gingiva, alveolar mucosa	Patchy erythematous macules or plaques with or without hyperplasia
Linear ulcerations	Vestibule, buccal mucosa, tongue, palate	Deep ulcerations with or without hyperplastic margins
Mucosal tags or polyps	Buccal mucosa, vestibule	Hyperplasia of mucosa, firm or boggy to palpation

Adapted from Kalmar [31].
